# Complete Genome Sequence of *Nocardia* sp. Strain CS682, a Producer of Antibacterial Compound Nargenicin A1

**DOI:** 10.1128/MRA.01098-19

**Published:** 2019-12-05

**Authors:** Dipesh Dhakal, Vijay Rayamajhi, Hue Thi Nguyen, Purna Bahadur Poudel, Jae Kyung Sohng

**Affiliations:** a Department of Life Science and Biochemical Engineering, Sun Moon University, Asan-si, Chungnam, Republic of Korea; b Department of Pharmaceutical Engineering and Biotechnology, Sun Moon University, Asan-si, Chungnam, Republic of Korea; University of Maryland School of Medicine

## Abstract

*Nocardia* sp. strain CS682 is a rare actinobacterium with a promising ability to produce secondary metabolites such as nargenicin A1 (an effective antibacterial compound) and IBR-3 (a UV-protectant molecule). Here, we report the complete genome sequence of *Nocardia* sp. CS682, obtained by PacBio sequencing as a single contig with 8,919,230 bp (GC content, 63.3%).

## ANNOUNCEMENT

*Nocardia* spp. are rare actinobacteria that are generally characterized as a source of diverse biomolecules with antibacterial, antifungal, cytotoxic, and immunosuppressive activities (1, 2). The major secondary metabolite derived from *Nocardia* sp. strain CS682 is nargenicin A1, an antibacterial polyketide with promising activity against various Gram-positive pathogenic bacteria (3). Similarly, IBR-3, a novel tetrahydroxynaphthalene (THN) derivative with UV protection effects, was also isolated from strain CS682 (4).

Strain CS682 was isolated from soil samples from Jeonnam, Republic of Korea, while screening for bacterial strains producing potential antibiotics against methicillin-resistant Staphylococcus aureus (MRSA) (3). For genomic extraction, strain CS682 was cultured in brain heart infusion (BHI) medium at 37°C and 200 rpm for 5 days (5). The genomic DNA of *Nocardia* sp. CS682 was isolated and purified using the TIANamp bacterial DNA kit. A PacBio SMRTbell library was constructed and sequenced on the PacBio RS platform (Macrogen, Inc., Republic of Korea) (6, 7). The Hierarchical Genome Assembly Process (HGAP) algorithm was executed in the Web-based graphical user interface (GUI) in the SMRT Analysis pipeline version 2.3.0 for preparing the PacBio library and for high-quality *de novo* assembly of the genome from PacBio raw reads. There were 152,943 sequence reads with a mean subread length of 9,897 bp, resulting in 189-fold sequencing coverage of the genome. Furthermore, a consensus sequence of higher quality was generated by validating the mapping reads against assembled contigs and error correcting using Quiver version 2.1.0 (8). Finally, the CS682 genome was assembled into a single contig. The genome was annotated with the NCBI Prokaryotic Genome Annotation Pipeline (PGAP) version 4.5 (9). The autoMLST pipeline (https://automlst.ziemertlab.com/download) (10) was used for determining closely related genomes based on alignment of core genes, and the closest species were determined based on percent average nucleotide identity (ANI). For determining the biosynthetic gene clusters (BGCs) in the genome, antiSMASH bacterial version 4.0 (11) was used. Default parameters were used for all software unless otherwise specified.

The assembled genome of strain CS682 was obtained as a single contig with 8,919,230 bp (GC content, 63.3%). A total of 7,856 open reading frames (ORFs), 56 tRNAs, 3 rRNA genes (5S, 16S, and 23S rRNA), and 3 noncoding RNA (ncRNA) genes were identified (Table 1). The estimated ANI values of Nocardia sp. CS682 with different *Nocardia* spp. were 86.7% (Nocardia altamirensis NBRC 108246; RefSeq assembly accession no. GCF_001612685), 86.7% (Nocardia brasiliensis ATCC 700358; GCF_000250675), and 86.7% (Nocardia brasiliensis NBRC 14402; GCF_000308475). These observations establish that *Nocardia* sp. CS682 has higher similarities to Nocardia altamirensis and Nocardia brasiliensis than to other species. The antiSMASH 4.0 results revealed that strain CS682 contains 44 putative BGCs, including clusters for polyketides, nonribosomal peptides, ectoine, terpenes, etc. The genomic architecture of strain CS682 also provided insight on precise locations of BGCs of nargenicin A1 (2) and IBR-3 (3) within the genome.

**TABLE 1:** Major genomic features of *Nocardia* sp. CS682

Genomic feature	Data
Genome size (bp)	8,919,230
GC content (%)	67.3
Contig *N*_50_ (bp)	14,297
No. of reads	152,943
No. of predicted ORFs	7,856
No. of predicted tRNA genes	56
Total no. of predicted rRNA genes (5S, 16S, and 23S)	3
No. of predicted pseudogenes	182
No. of predicted ncRNA genes	3
No. of predicted CRISPR arrays	4
BioProject identifier	PRJNA473642
BioSample identifier	SAMN09280253
Sequence Read Archive accession no.	SRX6973800, SRX6973801, and SRX6973802
GenBank genome accession no.	CP029710
No. of gene clusters predicted by antiSMASH	44

### Data availability.

The genome sequence of *Nocardia* sp. strain CS682 can be accessed in the NCBI database under accession no. CP029710. The PacBio raw reads have been submitted to the Sequence Read Archive (SRA) under accession no. SRX6973800, SRX6973801, and SRX6973802.
